# Exploring the Needs of People With Chronic Low Back Pain and Health Care Professionals for mHealth Devices to Support Self-Managed Physical Activity and Pain: User-Centered Design Approach

**DOI:** 10.2196/59897

**Published:** 2024-11-07

**Authors:** Mathilde Berger, Anne Deblock-Bellamy, Laurence Chèze, Thomas Robert, Julie J Desrosiers, Guillaume Christe, Anne Martine Bertrand

**Affiliations:** 1 Department of Occupational Therapy University of Applied Sciences and Arts Western Switzerland (HETSL | HES-SO) Lausanne Switzerland; 2 LBMC Claude Bernard Lyon 1 University Gustave Eiffel University Bron France; 3 Department of Physiotherapy School of Health Sciences (HESAV) University of Applied Sciences and Arts of Western Switzerland (HES-SO) Lausanne Switzerland

**Keywords:** chronic low back pain, needs, self-management, physical activity, mobile health, mHealth, user-centered design

## Abstract

**Background:**

Chronic low back pain (CLBP) is a major economic and social problem worldwide. Despite the variety of recommended treatments, long-term self-management of this condition is complex and requires the development of innovative interventions. Mobile health (mHealth) technologies hold great promise for the management of chronic pain, particularly to support physical activity. However, their implementation is challenged by a lack of user compliance and limited engagement, which may be due to insufficient consideration of the needs of potential users during development.

**Objective:**

This study aims to explore the needs of people with CLBP and health care professionals regarding mHealth technologies to support self-managed physical activity, and to delineate design recommendations based on identified needs.

**Methods:**

A participatory study was conducted using a 3-phase, user-centered design approach: needs investigation with a group of experts in a workshop (phase 1), needs exploration with end users in focus groups (phase 2), and validation of needs using Delphi questionnaires followed by the development of a set of recommendations (phase 3).

**Results:**

A total of 121 people with CLBP, expert patients, health care professionals, rehabilitation researchers, and biomechanical engineers participated in this study. The results indicated how technology could help people with CLBP overcome their difficulties with managing physical activity. Specific needs were formulated concerning device objectives, expected strategies, functionalities, technical features, conditions of use, and potential facilitators and barriers to use. These needs were validated by consensus from the potential end users and translated into design recommendations.

**Conclusions:**

This study provides design recommendations for the development of an mHealth device specifically adapted for people with CLBP.

## Introduction

### Background

Chronic low back pain (CLBP) is a major public health concern worldwide because of its economic and social consequences [1,2]. It is the leading cause of disability and work absenteeism, and its prevalence is still increasing [3,4]. Management of daily symptoms and activity are major challenges for people with this condition [5-7].

Self-management strategies and maintaining an active lifestyle have been consistently recommended for people with CLBP [8-12]. Self-management can be defined as the ongoing and dynamic ability to handle symptoms, such as pain, physical and psychological consequences, and lifestyle adjustments [13,14]. Self-management programs include educational and psychosocial interventions; maintaining an active lifestyle is a core component of these programs. People with CLBP are encouraged to engage in regular physical activity and to adopt healthy behaviors to manage their symptoms [14]. Although self-management and physical activity–based interventions improve pain [15] and reduce disability [16,17] in the long term, as well as promote the development of self-management skills [18,19], the effect of such interventions are small to moderate. Moreover, it is difficult to support self-management in clinical practice because of constraints in time, service organization, and follow-up [20]. Therefore, there is a need to identify innovative interventions to promote sustainable self-managed physical activity in people with CLBP.

Recent advances in technology like mobile health (mHealth) may offer new opportunities to support self-managed physical activity and pain in people with CLBP. mHealth is defined as a “medical and public health practice supported by mobile devices, such as mobile phones, patient monitoring devices, personal digital assistants, and other wireless devices” [21]. It can help people change their health behaviors [22] and help people with CLBP pursue specific physical activity goals while receiving continuous feedback on their physical performance [23,24]. It could be used to help them manage their condition and maintain an active lifestyle [25,26]. From the point of view of health care professionals, mHealth technology can provide individualized interventions with real-time feedback, support their coaching role [27], and support the development of behavior change [28,29].

Although mHealth technologies are promising, their content and the context in which they are offered need to be explored further. Many mobile apps are available to support people with CLBP in self-management, mainly providing exercise recommendations or information about the mechanisms of CLBP. These apps score poorly on the Mobile Application Rating Scale (a scale to assess app quality), and do not mention the theoretical approaches on which they are based [30-32]. Sometimes these mobile apps are combined with a physical activity tracker; however, they have shown limited effectiveness in disability and pain management in people with CLBP [33,34]. For example, several studies using the Fitbit device found nonsignificant results for pain [35,36] and disability [37]. This lack of an effect could relate to the fact that the target population for the tool is healthy individuals. Devices, such as the Fitbit were developed primarily to help young people improve their physical condition and they do not specifically consider people with CLBP and their context. They do not appear to be suitable for supporting health care professionals or people with CLBP who may benefit from increasing their participation in unstructured physical activity, like walking, and reducing sedentary lifestyle habits [38-41]. Moreover, studies of rehabilitation programs that integrated mobile technology found mixed results because of participants’ lack of adherence to the wearable technology [35,36].

It is now widely recognized that user-centered design (UCD) approaches are needed to facilitate the development, acceptability, and implementation of mHealth technologies [42-45]. In the field of CLBP, such designs are seldom used and rarely rigorously applied [46], particularly in the initial stages of device design [47].

### This Study

The main aim of this study was to explore the needs of people with CLBP and health care professionals regarding the use of mHealth technologies to support self-managed physical activity and pain. The specific aims were to (1) investigate how technology could help people with CLBP overcome the difficulties of managing their own physical activity with an expert group, (2) explore the needs, experiences, and preferences of people with CLBP and health care professionals regarding technology to support self-managed physical activity, and (3) validate the identified needs and develop a set of mHealth technology design recommendations.

## Methods

### Design

**Figure 1:**
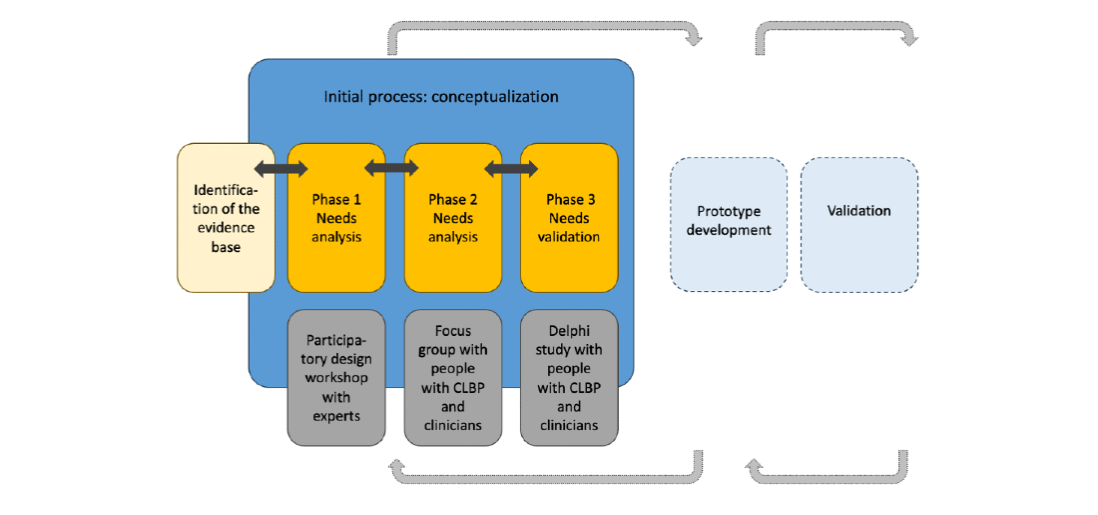
Illustration of the 3 phases of data collection based on the initial process of a user-centered approach. CLBP: chronic low back pain.

A participatory study was conducted using a UCD approach. UCD is an iterative process that focuses on users and their contexts in all stages of development design [44,48,49]. This study focused only on the initial process of the approach to investigate the needs of people with CLBP and to guide future prototype designs (Figure 1) [50-52]. The study was conducted in France and Switzerland and involved three successive data collection phases: (1) needs investigation with a group of experts, (2) needs exploration with people with CLBP and health care professionals, and (3) validation of the needs identified and development of recommendations. It was conducted after a preliminary literature review that aimed to identify existing evidence on mHealth devices used to support self-managed physical activity in people with CLBP.

### Ethical Considerations

The study was approved by the research ethics committee of the University of Lyon (CER-UDL 2022-10-13-003). The Cantonal Ethics Committee of Swissethics stipulated that this research was outside their jurisdiction and did not require ethics approval (Req-2022-00733).

### Data Collection

#### Phase 1: Needs Investigation With an Expert Group

#### Overview

The first phase investigated how technologies could support people with CLBP in overcoming difficulties with physical activity and pain self-management. A web-based participatory design workshop involving an expert group was set up for this purpose [50].

#### Sample

The expert group consisted of patients who were experts, clinicians with expertise in low back pain management, rehabilitation researchers, and biomechanical engineers in the rehabilitation field. Inclusion criteria for patients who were experts in self-management of their disease acquired over several years, and experience with several treatment modalities, including participation in at least 1 multidisciplinary rehabilitation program. Clinicians, rehabilitation researchers, and biomechanical engineers were required to have at least 5 years of experience in their respective fields.

#### Recruitment

The expert group was recruited through the investigators’ clinical networks. Potential experts were contacted via email and telephone by the research team who explained the workshop to them, invited them to participate, and obtained their consent.

#### Data Collection

Data were collected during a 3-hour web-based participatory design workshop via teleconference in October 2022, moderated by 2 members of the research team (AD-B and MB). The workshop was organized into a preliminary activity followed by 3 phases. Before the workshop began, each participant received a summary of the existing evidence on mHealth devices used to support self-managed physical activity and pain in people with CLBP. In the first part of the workshop, the demographic characteristics of the participants were collected using a short questionnaire. Each of the 4 expert subgroups (patients who were experts, clinicians, rehabilitation researchers, and biomechanical engineers) was asked to reflect on the difficulties and factors limiting self-managed physical activity and pain in people with CLBP. In the second part, 2 subgroups were formed with different expert representatives (each subgroup consisted of at least 1 patient who was an expert, 1 clinician, 1 rehabilitation researcher, and 1 biomechanical engineer) to encourage discussion and the generation of several ideas on how technology could help people with CLBP overcome the difficulties identified. Each subgroup explored potential solutions to address the difficulties identified for a described persona. All exchanges with the group were recorded and transcribed.

#### Phase 2: Needs Exploration With End Users

#### Overview

The second phase explored the needs, experiences, and preferences of people with CLBP and health care professionals for using technology to support self-managed physical activity and pain. Two series of focus groups were run for this purpose [51]. The first explored end-user experiences and needs relating to physical activity and pain self-management. The second focused on potential technological solutions to meet the identified needs.

#### Sample

A sample of people with CLBP and a sample of health care professionals were recruited. Inclusion criteria for people with CLBP were a clinical diagnosis of CLBP and participation in ongoing rehabilitation within a multidisciplinary program. Inclusion criteria for health care professionals were more than 2 years of experience caring for people with CLBP and participation in a multidisciplinary rehabilitation program for CLBP.

#### Recruitment

Recruitment was done in collaboration with 2 rehabilitation centers, one in Switzerland and the other in France. Health care professionals involved in the care of people with CLBP at both rehabilitation centers were informed of the study via an email from the research team with information about the study attached. People with CLBP were informed about the study by their health care professionals and received written information about the study. All people with CLBP and health care professionals willing to participate were contacted by the research team who provided further information and collected signed informed consent for participation. All participants included in phase 2 were different from those in phase 1 to explore diverse perspectives.

#### Data Collection

The 2 sets of focus groups were conducted separately for people with CLBP and health care professionals at the rehabilitation centers in Switzerland and France, with 4 to 8 participants as recommended by Kitzinger et al [51]. Each participant attended 2 focus groups held 2 weeks apart between January and February 2023. In the first series of focus groups, participants shared their experiences and needs regarding self-managed physical activity and pain in relation to the issues outlined by the expert group. In the second series of focus groups, participants discussed the potential technological solutions to meet the identified needs, in detail. A total of 8 focus groups were conducted by the research team. Participants were asked about their past experiences with health care technologies and their potential future needs for using technology. The potential facilitators and barriers to using technology to support self-managed physical activity and pain were also discussed. Two members of the research team moderated all the focus groups using a standardized interview guide developed by the research team (MB, JJD, or AD-B). An interview guide was developed for people with CLBP and health care professionals, asking the same questions but rephrased specifically for each group. Each focus group lasted approximately 1 hour 30 minutes and was recorded and transcribed. Participant’s demographic characteristics were collected using a short questionnaire before the focus groups, and all data were coded to ensure anonymity.

#### Phase 3: Needs Validation

#### Overview

The third phase involved validating the identified needs and delineating a set of design recommendations for mHealth technologies. For this, a two-round Delphi questionnaire approach was used [52]. The first round assessed the needs expressed by participants during the previous consultation phases, and the second round consisted of evaluating the responses that did not reach a consensus based on the suggestions from the first round.

#### Sample

People with CLBP and health care professionals were selected. The inclusion criterion for people with CLBP was having already completed or in the process of completing a multidisciplinary rehabilitation program. The inclusion criterion for health care professionals was more than 2 years’ experience caring for people with CLBP.

#### Recruitment

Participants were recruited through poster advertising, social media, and word of mouth, in Switzerland and France. Participants from phase 2 were invited to join phase 3, allowing them to evaluate all the needs identified in the earlier phases, not just those discussed in their focus groups. All recruitment materials directed interested individuals to a web-based survey (LimeSurvey) to determine their eligibility, register their characteristics, and provide informed consent.

#### Data Collection

The Delphi questionnaire was developed from all the themes identified from the results of the 2 previous phases of the study. Two researchers (MB and AD-B) selected each statement for the questionnaire. The research team then validated the statements according to the elements discussed during the previous 2 study phases. Before completing this questionnaire, the research team provided participants with a standardized summary of the methodology and the preliminary results of the first 2 phases. All participants completed a short sociodemographic questionnaire. Each participant was asked to rate their level of agreement with each statement on a 4-point bidirectional scale: strongly disagree (1), disagree (2), agree (3), or strongly agree (4). If participants “disagreed” or “strongly disagreed,” they were invited to propose a new suggestion corresponding to their own opinion in open text boxes. The first round of questionnaires remained open for 4 weeks. Consensus was considered achieved if 90% of the participants agreed or strongly agreed with a statement. The second-round questionnaire was based on suggestions for statements for which there was no consensus in the first round. The second round was open for 12 weeks and only people who had already responded to the first round were invited to respond to the second round. This third phase lasted 16 weeks, from June to September 2023.

### Data Analysis

Descriptive statistics were used to characterize the participants in the 3 phases, using frequencies and percentages. In phase 1, participant discussions were recorded and transcribed verbatim for analysis using an inductive thematic analysis [53,54]. First, 2 researchers (MB and AD-B) independently read the transcripts to familiarize themselves with the content. Second, they separately coded the data using NVivo (version 20, Lumivero) to create an initial codebook. After the initial coding, the codebook was discussed between the 2 researchers, and segments of the content with similar meaning were assigned to the same code. Third, the coded transcripts were used to refine the concepts of the initial codebook and combine the codes into key themes. When new codes or themes emerged, the codebook was revised, and the previous transcripts were recoded. Any discrepancies between the researchers were resolved by discussion, and any necessary adjustments were made. This thematic analysis was then conducted again to reach a consensus. Finally, the content of the themes and subthemes of the coding scheme was discussed with the entire research group.

In phase 2, the data analysis procedure from phase 1 was repeated using recorded material from all the focus groups [53,54] and the field notes written by the moderator in charge of describing the group interactions. The final version of the codebook from phase 1 was reused and revised by 2 researchers for use in phase 2 (MB and AD-B). All themes were validated by the entire research group.

In phase 3, the level of expert consensus was calculated for each item after each round [55]. Consensus was considered achieved when 90% of the participants agreed or strongly agreed with a statement. All suggestions were reviewed by 2 researchers (MB and AD-B), and the data were coded using the same codebook used for phases 1 and 2.

## Results

### Overview

A total of 121 participants were involved in the study. Of the 121 participants, 9 experts took part in the first phase, including 4 subgroups of 2 to 3 experts in the first part of the workshop and 2 subgroups of 4 to 5 experts in the second part of the workshop (one of the subgroups was composed of 1 patient who was an expert, 1 clinician, 1 rehabilitation researcher, and 1 biomechanical engineer while the other was composed of 1 patient who was an expert, 2 clinicians, 1 rehabilitation researcher, and 1 biomechanical engineer). Of 26 participants who took part in the second phase, 11 people with CLBP and 15 health care professionals (including 4 and 7 participants, respectively, for the 2 sets of focus groups with people with CLBP, and 7 and 8 participants, respectively, for the 2 sets of focus groups with health care professionals). Of 86 participants who took part in the third phase, 45 people with CLBP and 41 health care professionals. About 63% (7/11) of the participants with CLBP and 46% (7/15) of health care professionals from phase 2, participated in phase 3. The demographic characteristics of the participants are presented in Table 1 and more details about the participants are available in Multimedia Appendix 1.

More than 12 hours of participatory design workshop data, focus group data, and suggestions from 2 questionnaires were coded into 5 themes that are described in more detail below: (1) difficulties experienced by people with CLBP in relation to self-managed physical activity and pain, (2) device concept (aim and strategies), (3) device content, (4) condition of use, and (5) facilitators and barriers to use.

**Table 1:** Characteristics of the participants in each phase of the study.

Participant characteristics		Phase 1 (n=9), n (%)	Phase 2 (n=26), n (%)	Phase 3 round 1 (n=86), n (%)	Phase 3 round 2 (n=61), n (%)
**Gender**					
	Women	6 (67)	16 (62)	46 (54)	36 (59)
	Men	3 (33)	10 (38)	39 (45)	24 (39)
	Other	0 (0)	0 (0)	1 (1)	1 (2)
**Age (y)**					
	20-29	0 (0)	1 (4)	8 (9)	3 (5)
	30-39	4 (44)	6 (21)	20 (23)	14 (23)
	40-49	0 (0)	9 (35)	17 (20)	12 (20)
	50-59	5 (56)	8 (31)	30 (35)	22 (36)
	≥60	0 (0)	2 (8)	11 (13)	10 (16)
**Role**					
	People with CLBP^a^	2 (22)	11 (42)	45 (52)	33 (54)
	Health care professional	3 (33)	15 (58)	41 (48)	28 (46)
	Rehabilitation researcher	2 (22)	—^b^	—	—
	Research engineer	2 (22)	—	—	—
**Location**					
	Switzerland	5 (56)	11 (42)	63 (73)	42 (69)
	France	4 (44)	15 (58)	23 (27)	19 (31)

^a^CLBP: chronic low back pain.

^b^Not applicable.

### Difficulties Encountered by People With CLBP in Relation to Self-Managed Physical Activity

The difficulties faced by people with CLBP in relation to self-managed physical activity and pain were specifically explored in phase 1 by the expert group composed of patients who were experts, clinicians, rehabilitation researchers, and biomechanical engineers. All participants were asked to discuss these difficulties to reach a consensus on the problems faced by people with CLBP. These difficulties were then iteratively addressed by people with CLBP and health care professionals in phases 2 and 3. These difficulties are presented in Table 2. The more prevalent ones included pain, personal beliefs, difficulty pacing physical activity, and a lack of sustained, individualized, long-term support from health care professionals. Only these prevalent difficulties were used as the basis for the assessment of the needs of people with CLBP and health care professionals, as they were shared by both groups.

**Table 2:** Difficulties and factors limiting self-managed physical activity and pain in people with chronic low back pain (CLBP) identified by the different groups of participants in the different phases.

Difficulties reported	Phase 1					Phase 2			Phase 3	
	eP^a^	eHp^b^	Rr^c^	Re^d^	People with CLBP		Hp^e^	People with CLBP		Hp
Chronic pain	✓^f^	✓	✓	✓	✓		✓			
Personal beliefs: fear of pain and underlying injury, kinesiophobia, difficulty understanding that the activity can be beneficial, loss of purpose, etc	✓	✓	✓	✓	✓		✓	✓		
Difficulty pacing physical activity	✓	✓	✓		✓		✓			
Sedentary lifestyle and lack of time	✓	✓	✓		✓		✓	✓		
Lack of long-term support	✓	✓			✓		✓	✓		
Lack of individualized support	✓		✓		✓			✓		
Poorly coordinated health care pathway		✓	✓				✓			
Lack of motivation				✓	✓		✓			
Negative experiences with physical activity	✓		✓		✓					
Difficulty making long-term plans	✓				✓		✓			
Beliefs of those around you and of society in general		✓	✓							
Depression, loss of confidence in one’s own abilities		✓		✓						
Neurophysiological damage		✓								
Socioprofessional and safety problems		✓								
Medication side effects		✓								
Body image disorders		✓								
Feeling overwhelmed		✓					✓			
Fatigue, sleep disorders		✓								
Eating disorders		✓								
Laziness		✓								
Frustration with one’s own abilities					✓					
Forgetting the principles of activity management							✓			

^a^eP: patient who was an expert.

^b^eHp: expert health care professional.

^c^Rr: rehabilitation researcher.

^d^Re: research engineer.

^e^Hp: health care professional.

^f^Difficulty mentioned by the group.

### Device Concept

The needs of people with CLBP and health care professionals regarding the device concept are described in Tables 3 and 4 and illustrated with verbatim. All participants highlighted the importance of having a device that would not only help increase physical activity level of people with CLBP but also better pace it. They insisted on the need for this device to also help people with CLBP with long-term pain management. To achieve these objectives, they suggested the device should support the following behavior change strategies: goal setting, self-monitoring, feedback, positive reinforcement, reward delivery, and social support.

**Table 3:** Needs relating to device concept, especially objectives of the device, identified by experts, people with chronic low back pain (CLBP), and health care professionals.

Needs identified	Quotes (translated from French into English)
Help to pace physical activity	“[The device should] allow self-pacing.” [Person with CLBP; phase 2] “I want it to be regular...even if performance increases progressively, the fact that it’s regular is precisely to maintain the benefits for the back in the long term.” [Person with CLBP; phase 2]
Support long-term self-management of pain and not only physical activity	“That it’s not just a device to improve physical activity, but also symptom management, pain management.” [Ieg^a^; phase 1] “The device must really improve the patient’s well-being.” [Ieg; phase 1]

^a^Ieg: interdisciplinary expert group.

**Table 4:** Needs relating to device concept, especially expected behavior change strategies supported by the device, identified by experts, people with chronic low back pain (CLBP), and health care professionals.

Needs identified	Quotes (translated from French into English)
Goal setting	“What seemed important was that this device already allowed a certain degree of individualization, for example, based on patient goals.” [Ieg^a^; phase 1] “There must be clear goals. Without clear objectives, we’ll just keep going. We’ll get the story we want.” [Person with CLBP; phase 2]
Self-monitoring	“The system should allow the patient to monitor their progress.” [Ieg; phase 1] “To move forward, you need to know what’s happened recently. How can you know if you don’t have any data? Yes, it’s been the same for 6 months, but the same, better, less? Well, I don’t know. I can’t remember.” [Person with CLBP; phase 2]
Feedback and coaching advice sent by the device	“We could also imagine motivational alerts if you’ve walked a little less or have been a little less active in the last few days. Not in a negative way, but in a positive way.” [Ieg; phase 1] “To have that on the smartphone to say, ‘Ah today you basically did what you needed to do.’ Or at the end of the day ‘you haven’t done all your steps’ or ‘you’re missing some targeted exercises’ and that would be magic.” [Person with CLBP; phase 2]
Positive reinforcement	“It’s important that the words are kind and positive. You don’t want to hear, ‘Are you in pain?’” [Person with CLBP; phase 2] “I think it’s better to take into account positive emotions and parameters than to point out negative parameters such as stress!” [Hp^b^; phase 3]
Reward delivery	“We wondered if something a little bit like a game could also stimulate activity...if the device could collect points or have something to help motivate to do exercises.” [Ieg; phase 1] “You took longer, you did more’ suits me well, I’ve won stars, I’ve changed levels, super, I’m super happy. I mean, it’s working really well for me.” [Person with CLBP; phase 2]
Education	“We know that when it comes to pain; simply put, the more you know, the less pain there is. So I don’t know if a questionnaire that picks up things that have been covered in a program...to see if people still remember them, are able to remember them.” [Hp; phase 2] “To maintain our level of knowledge. All knowledge, in the end, we tend to go back to the representations we had in the past.” [Hp; phase 2]
Social support through testimonials	“Maybe if you can have patient testimonials, examples of people who have found solutions or things that are difficult and then they’ve been able to solve them. Maybe that can be motivating.” [Ieg; phase 1] “People with low back pain sometimes suffer from negative images, and to see that others have been successful, in quotes, I think can motivate them, even if it’s anonymous.” [Hp; phase 2]

^a^Ieg: interdisciplinary expert group.

^b^Hp: health care professional.

### Device Content

The needs of people with CLBP and health care professionals regarding device content are described in Tables 5 and 6 and illustrated with verbatim. Participants expressed the need for a device that would allow people with CLBP to automatically track the following data: number of steps, heart rate, activity versus rest time, intensity of the activity, stress, and sleep. They emphasized the need for the collection of additional data recorded by the user, such as an activity diary, well-being, pleasure, satisfaction, comfort, medications taken, pain, sleep, and stress. They insisted that the user should specifically select automatically-collected and self-reported data. Participants also wanted the device to be able to send notifications to help people with CLBP manage their physical activity and pain. They highlighted the usefulness of receiving regular activity reports and of being able to access specific resources, such as physical exercises, relaxation exercises, and questionnaires to refresh knowledge about pain. They suggested the use of a wristband combined with a digital app that is easy to use, discreet, comfortable, waterproof, and robust.

**Table 5:** Needs relating to device content, especially functionalities, identified by experts, people with chronic low back pain (CLBP), and health care professionals.

Needs identified	Quotes (translated from French into English)
Automatically and effortlessly collect the user’s activity data: steps, heart rate, active-resting time, intensity of activity, stress, and sleep	“I think, on the contrary, to be able to objectively go back to your daily activities and then read, ‘Oh well, yes, actually I’m doing a lot more than I feel I’m doing’ or ‘I’m doing a lot less than I feel I’m doing,’ that’s important.” [Hp^a^; phase 2]
Recording of data entered by the user: activity diary, well-being, pleasure, satisfaction, comfort, medication taken, pain, stress, and sleep	“To be able to quantify pain.” [Person with CLBP; phase 2] “All measures, obviously pleasure activities.” [Hp; phase 2] “I am not in favor of focusing on pain, but on the positive management of pain, what is the point of quantifying it for the sake of quantifying it, the nocebo vocabulary is still used too much, and everything is still based too much on negative criteria.” [Hp; phase 3, round 2]
Notifications, alerts, and messages tailored to the user	“If we think about artificial intelligence, we can imagine that the application will gradually be able to personalize advice based on the data collected.” [Ieg^b^; phase 1] “It could say: ‘You’re too stressed, do what’s necessary to reduce it.’” [Person with CLBP; phase 2]
Reports: history accessible over a variable period (day, week, and month)	“Have a PDF report of what we do at the end.” [Person with CLBP; phase 2] “The device captures all this data and then we make a summary to get an overview over several days, weeks, or even months.” [Hp; phase 2]
Information transfer: provision of personalized physical exercises, provision of relaxation exercises, and provision of pain reminder questionnaires	“I think with targeted exercises, but clearly individually, because we all do, we all have different activities.” [Person with CLBP; phase 2] “I’ve used a lot of digital tools, meditation tools, instant meditation support tools. It’s been a great help.” [Person with CLBP; phase 2] “We tend to go back to the representations we had in the past. Questionnaire reminders.” [Hp; phase 2]
Personalization: initial settings for automatically-collected data and data entered by the user, setting an activity goal	“It’s important to be able to personalize all the elements that the patient sees in a visual format or personalize them according to what they want to see.” [Ieg; phase 1] “Adapt the measured data.” [Ieg; phase 1] “Any personalization, tracking, and visualization options are welcome. The important thing is to give patients the freedom to choose what they want to track and share.” [Person with CLBP; phase 3]

^a^Hp: health care professional.

^b^Ieg: interdisciplinary expert group.

**Table 6:** Needs relating to device content, especially characteristics, identified by experts, people with chronic low back pain (CLBP), and health care professionals.

Needs identified	Quotes (translated from French into English)
Support: wristband combined with a digital app on a smartphone	“Just a wristband can be more discreet, with just one sensor. And then on your phone you’ll see what you want to see.” [Person with CLBP; phase 2]
Ease of use	“For me, the keyword in all of this is simplicity. In all that it can mean in terms of tools, in terms of use, in terms of presentation. I really mean simplicity in the broadest sense.” [Ieg^a^; phase 1] “Something that’s hyper user friendly, because otherwise it’s not worth it.” [Person with CLBP; phase 2]
Esthetics: discreet, comfortable, attractive, different colors, waterproof, and robust	“In terms of comfort, something that’s light, that’s flexible.” [Person with CLBP; phase 2] “It must also be an attractive object.” [Hp^b^; phase 2] “The device would have to be waterproof because swimming is good for my pain and it would be a shame to lose my physical activity data. Also, if you’re going to wear it 24 hours a day, it needs to recharge quickly.” [Person with CLBP; phase 3]

^a^Ieg: interdisciplinary expert group.

^b^Hp: health care professional.

### Conditions of Use

The needs of people with CLBP and health care professionals regarding the conditions of use are described in Table 7 and illustrated with verbatim. Participants indicated that this device may be used either 24 hours per day or only during the day, depending on the user’s preference. They wanted it to be provided by a health care professional as part of a rehabilitation program, and they wanted health care professional supervision for its use. They wanted to be able to consult the data collected in real time, but some health care professionals expressed concern about misinterpretation and suggested that the data should initially be only available in the presence of a therapist. All participants insisted on the importance of long-term follow-up by a health care professional to check the use of the device and to help adjust the goals (either face-to-face or by videoconference).

**Table 7:** Needs relating to conditions of use identified by experts, people with chronic low back pain (CLBP), and health care professionals.

Conditions of use	Needs identified	Quotes (translated from French into English)
Frequency of use of the device	Can be used continuously all day, or night and day (according to the user’s needs)	“It has to be worn 24/7.” [Person with CLBP; phase 2] “I’d say all day, 24 hours a day.” [Hp^a^; phase 2] “Be careful about wearing 24/7—to be determined according to the objectives and the patient—I think wearing it all the time is difficult over time too.” [Hp; phase 3]
Conditions for providing the device	By a health professional, as part of a rehabilitation program, after educational therapy	“The devices are not that easy to use, so it’s also part of the therapist’s role to teach how to use them, or even provide tutorials.” [Ieg^b^; phase 1] “Something that will be implemented during therapeutic education workshops when we’re doing nondrug tools.” [Hp; phase 2]
Conditions for consulting the data for the user	In real time or when the user is a novice: possibly accompanied by a health care professional the first few times	“You go to the app, you look when you feel like it.” [Person with CLBP; phase 2] “That they (patients) have access to information a posteriori, that they don’t have access to information in real time, that is, that the watch or eventually the device. The application will record the data, but it won’t be visible. It will only be visible after x amount of time.” [Hp; phase 2] “Real time also has its advantages. It can be motivating, depending on the parameters. There’s the whole biofeedback side, which has proven to be very useful in certain situations.” [Hp; phase 2]
Follow-up conditions related to device use	Follow-up session with a health care professional after a period of device use, possibly by videoconference	“And maybe the digital tool and, at some point, a human relay that comes back on time that we can readjust.” [Person with CLBP; phase 2] “That’s when I tell myself that teleconsultation makes sense, even in a group. At the end of a program or afterward, when you’ve really got an appointment.” [Hp; phase 2] “To have a regular appointment with a professional to take stock.” [Person with CLBP; phase 3] “For it to be beneficial, you’d have to have a regular review with a health professional, readjust the objectives.” [Person with CLBP; phase 3]

^a^Hp: health care professional.

^b^Ieg: interdisciplinary expert group.

### Facilitators and Barriers to Device Use

The facilitators and barriers to the use of a device mentioned by the participants in all the study phases are described in Table 8. The facilitators mentioned corresponded to the functionalities and features considered necessary for the content of the device. The potential barriers were related to concerns about the validity of the data collected, the storage and confidentiality of that data, the need to recharge the device, the additional availability of health care professionals to provide and monitor the device, and the risk of adverse effects associated with the use of the device.

**Table 8:** Facilitators and barriers to the use of a device identified by people with chronic low back pain (CLBP) and health care professionals in phases 2 and 3.

Facilitators and barriers		Quotes (translated from French into English)
**Facilitators**		
	Personalization	Provided in Table 5
	Ease of use	Provided in Table 6
	Esthetic	Provided in Table 6
	Conditions of use	Provided in Table 7
**Barriers**		
	Validity of automatic data collection	“(The device) is not ready for sleep yet.” [Person with CLBP; phase 3] “In addition, the measurement accuracy of these objects is still very random.” [Hp^a^; phase 2] “Heart rate is a very interesting variable, but the measuring devices are often unreliable.” [Hp; phase 3]
	Data storage and security	“I’m not so much a fan of data in the cloud. I’m not a big fan of data that just magically disappears.” [Person with CLBP; phase 2] “To have this tool that measures all our activities for weeks on end? Well, there are things that are a little bit in the realm of privacy that will also be measured.” [Person with CLBP; phase 2]
	Instrument: setting up and reloading the device	“The debate is about the battery and recharging.” [Person with CLBP; phase 2] “The only drawback, like any electronic device, is that it needs to be recharged, which creates a time lapse where the data is not, cannot be collected automatically and subjectively by the device.” [Hp; phase 2]
	Conditions for making the device available and monitoring its use	“In the feasibility of the current programs. We’d need more time to integrate such a tool, because if we have to add things. If we have to add another tool to integrate. On top of what we’re already asked to do. Of course, that means extra time.” [Hp; phase 2]
	Adverse reactions linked to the use of the device: misinterpretation, guilt	“They make cause-and-effect relationships that are very random. And these objects also have a very random precision of measurement, so sometimes they come to conclusions that are completely off the mark, and sometimes, in my opinion, in some cases, I’m not saying for all, but it can be counterproductive.” [Hp; phase 2] “Wearing a connected bracelet 24 hours a day can be stimulating for some profiles, but it can also be guilt-inducing for others.” [Hp; phase 3]

^a^Hp: health care professional.

### Needs Validation

The needs of people with CLBP and health care professionals regarding device concept and content and the conditions of use were confirmed in 2 rounds of Delphi questionnaires with 86 and 61 participants, respectively (Tables 9 and 10). Twenty-eight needs were selected from those identified in the previous 2 study phases. A lot of attention was given to the variables that could be collected or recorded by the device, as much of the discussion in phases 1 and 2 focused on these aspects. In the first round, consensus was reached on 13 (46%) of the 28 needs (the 13 validated needs are italicized in Table 9). In the second round, the 15 needs for which there was no consensus were modified based on the suggestions made in the first round of consultation: 11 (73%) of the 15 needs were reformulated, and 3 (20%) of the 15 needs were merged into a single need (changes in needs are italicized in Table 10). In the second round, consensus was reached on 11 (91%) of the 12 needs. Out of 25 needs in total, a consensus was reached on 24 (96%) needs.

**Table 9:** Degree of consensus on the items proposed in the Delphi questionnaire for each participant group (round 1).

Items: “The device should make it possible to”	All, (N=86; %)	People with CLBP^a^ (n=45; %)	Hp^b^ (n=41; %)
*Achieve a personalized physical activity goal* ^c^ *(Cc* *^d^)*	99	98	100
*Produce a report on changes in the user’s physical activity^c^ (Ct* *^e^)*	99	98	100
*Be set up with the support of a Hp as part of a rehabilitation program^c^ (Cu* *^f^)*	98	98	98
*Provide various pain management tools and techniques (eg, relaxation, cardiac coherence, etc)^c^ (Ct)*	97	98	95
*Generate a report of the variables collected that can be given to a Hp^c^ (Cu)*	97	96	98
*Assess and monitor the physical performance of the user^c^ (Cc)*	95	96	95
*Quantify heart rate automatically and in real time^c^ (Ct)*	93	100	85
*Quantify the time spent on activities of different intensities automatically and in real time^c^ (Ct)*	93	98	88
*Qualify the quality of sleep at a set frequency (eg, NS *^g^*: 0-10)^c^ (Ct)*	93	98	88
*Qualify the activities carried out at a set frequency^c^ (Ct)*	93	98	88
*Quantify activity and rest periods automatically and in real time^c^ (Ct)*	93	96	90
*Quantify the sleep duration automatically and in real time^c^ (Ct)*	92	96	88
*Monitor physical performance according to variables chosen by the user^c^ (Ct)*	91	98	83
Display changes in the user’s activity in real time (Cu)	90	96	83
Qualify satisfaction at a set frequency (eg, NS: 0-10) (Ct)	88	93	83
Be offered on an optional basis, as a complement to the rehabilitation (Cu)	88	93	81
Qualify level of stress at a set frequency (eg, NS: 0-10) (Ct)	87	96	78
Consist of a connected wristband combined with a digital application (Ct)	87	90	85
Qualify the level of pleasure at a set frequency (eg, NS: 0-10) (Ct)	86	91	81
Qualify pain intensity at a set frequency (eg, NS: 0-10) (Ct)	85	96	73
Quantify the number of steps taken automatically and in real time (Ct)	84	96	71
Qualify feelings at a set frequency (eg, NS: 0-10) (Ct)	84	91	73
Qualify “weather emotions” at a set frequency (eg, by ticking the weather image for actual feeling) (Ct)	84	84	83
Quantify stress levels automatically and in real time (eg, automatic score based on heart rate variability) (Ct)	83	96	76
Qualify medication taken at a set frequency (eg, type and dose) (Ct)	81	89	73
Obtain rewards (eg, badges or points) (Cc)	81	76	88
Be available, on an optional basis, after the end of the rehabilitation (Cu)	78	96	61
Be wearable 24 hours a day (Cu)	66	78	54

^a^CLBP: chronic low back pain.

^b^Hp: health care professionals.

^c^Validated needs are italicized.

^d^Cc: concept device.

^e^Ct: content device.

^f^Cu: condition of use.

^g^NS: numeric scale.

**Table 10:** Degree of consensus on the items proposed in the Delphi questionnaire for each participant group (round 2).

Items modified according to the results of the first round (changes are italicized). The device should be able to...	All (N=61; %)	People with CLBP^a^ (n=33; %)	Hp^b^ (n=28; %)
Consist of an object, such as a *connected watch* or bracelet combined with a digital application (Ct^c^)	100	100	100
Qualify “weather emotions” at a set frequency *if the user wishes* (eg, by ticking the weather image representing actual feelings). *The choice to take this data into account may be discussed with a Hp* (Ct)	98	100	96
Be offered on an optional basis, as a complement to the rehabilitation program, *by a Hp* (Cu^d^)	98	100	96
Be wearable every day *during the day,* or 24 hours a day *(depending on the data the user wishes to collect)* (Cu)	98	97	100
Qualify *the pain treatments used, if the user wishes,* at a set defined frequency (eg, the *type and dose of medicinal treatments, the type of stretching and duration, breathing techniques, and the time taken to perform them). The choice of taking this information into account may be discussed with a Hp* (Ct)	97	100	93
See activity progression in real time, *if the user wishes. It could also enable users to see how their activity is progressing at certain stages in their support, in partnership with a Hp involved in the rehabilitation program* (Cu)	97	100	93
Quantify the number of steps taken automatically and in real time. *The choice of how this data is taken into account may be discussed with a Hp* (Ct)	97	100	93
Qualify, at a set frequency, a *parameter with a positive connotation,* such as satisfaction, pleasure, or feeling, *if the user wishes. The choice of how this data is taken into account could be discussed with a Hp* (Ct)	97	97	96
Qualify according to a set frequency the level of stress*, if the user wishes* (eg, NS^e^: 0-10). *The choice of how this data is taken into account may be discussed with a Hp* (Ct)	95	97	93
Qualify the intensity of the pain at a set frequency, *if the user wishes* (eg, NS: 0-10). *The choice of how this data is taken into account may be discussed with a Hp* (Ct)	93	100	86
*Have an attractive “challenge” section* (eg, with a badge or points system), *that the user would be free to consult or not, to motivate him or her* (Cc^f^)	92	91	93
Be available, on an optional basis, after the end of the rehabilitation program, *depending on the person’s degree of motivation to use it and their desire to make a financial investment in the device* (Cu)	89	100	75

^a^CLBP: chronic low back pain.

^b^Hp: health care professionals.

^c^Ct: content device.

^d^Cu: condition of use.

^e^NS: numeric scale.

^f^Cc: concept device.

### Synthesis of Design Recommendations

**Figure 2:**
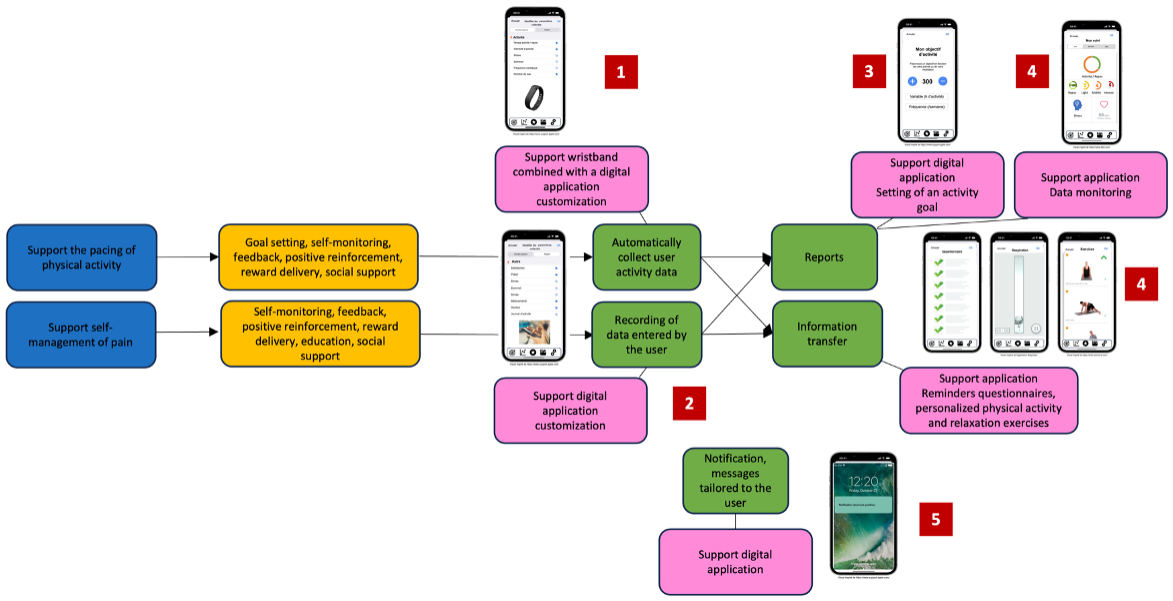
Illustration of design recommendations for a mobile health device that specifically meets the identified needs of people with chronic low back pain and health care professionals to support self-managed physical activity.

The needs validated by the participants were synthesized as design recommendations using the Behavioral Intervention Technology model and are illustrated in Figure 2. This is based on the illustration of the MyFitnessPal mHealth app by Mohr et al [56] and describes the expected functionalities and technical features of the device. The numbers shown illustrate the conditions of use and the expected workflow: (1) first, the automatic data collected by the device can be selected, then (2) the data collected by the user can also be selected. From this, (3) an activity objective can be defined, (4) and the user can follow changes in the values of variables collected or recorded, referring, if he or she wishes, to the physical or relaxation exercises proposed. (5) Reception of regular notifications and alarms can be set.

## Discussion

### Principal Findings

This study explored the needs of people with CLBP and health care professionals regarding the use of mHealth technology to support self-managed physical activity and pain. It elucidated how people with CLBP believed the adoption of an mHealth device could mitigate their difficulties in managing physical activity in relation to their pain, beliefs, difficulties in pacing physical activity, and the need for long-term individualized support.

The results of this study show that participants (people with CLBP and health care professionals) expressed interest in using an mHealth system to help people with CLBP manage their physical activity and pain over the long term. They wanted a device that would allow them to set personalized activity goals, monitor their activity, receive feedback on their performance, receive positive reinforcement, possibly receive rewards, learn information, and receive social support. These results provide a more precise definition of the needs identified by Merolli et al [46], who highlighted the usefulness of developing a technological solution to support the self-management of CLBP with tracking, notifications, feedback, provision of educational resources, and exercises. These results, collected using a rigorous process, accurately describe the expectations of end users in terms of the characteristics of a potential device.

Special attention was paid to the functionalities and variables recorded by the device. This is a fundamental step in the development of effective technology [57]. People with CLBP and health care professionals described the need to set realistic activity goals and to track them using an mHealth device, while also having complementary tools to help them manage their pain daily (eg, a physical activity exercise bank and relaxation exercises). They strongly emphasized that physical activity should be monitored using self-selected variables, stating that the need was not always to increase physical activity level, but rather to better pace it daily through achievable goals. Several participants with CLBP highlighted their tendency to overdo activity, debunking the assumption that physical activity levels in people with CLBP are often lower than in healthy people [58-60].

Overall, people with CLBP and health care professionals expressed similar needs in terms of an mHealth device. They strongly emphasized that the settings should be personalized, and the device should be easy to use. However, the opinions of these groups differed regarding the variables that should be collected and the components that should be provided by the device. Health care professionals believed the focus should not be on the assessment of negative variables, such as pain, whereas people with CLBP wanted to collect this type of variable. Health care professionals suggested that a “challenge” module should be included, with a system to collect points or badges, to encourage users to interact with the device. The opinions of the people with CLBP were more divided about such a module, with some very interested in this type of reward and others completely opposed to it because they were afraid of being involved in competitive challenges. The gamification of mHealth is attracting increasing interest as a means of encouraging changes in physical activity behavior, although current evidence on the effectiveness of this type of device is still limited [61,62].

The potential disadvantages and adverse effects associated with the use of a device to self-manage physical activity were discussed in detail during the 3 phases of the study. On the one hand, the effects of such a device on the development of new behaviors and lifestyles may be directly linked to its maintenance. For example, behavior change is less likely to occur if the device monitor is lost, broken, not charged, or forgotten. In contrast, if the device is used consistently and integrated into an intervention aimed at maintaining new behaviors, new habits could emerge in the medium term [63,64]. In addition, some health care professionals were concerned that people with CLBP could misinterpret the data collected by the device, and that this could induce feelings of guilt. Indeed, some health care professionals were against users being able to consult their data in real time, whereas people with CLBP saw this as an obvious possibility. The ability of the mHealth device to provide feedback to the user is considered important for encouraging behavioral change [61,65]. Health care professionals also mentioned the additional time required to teach their patients to use the device. In addition, both health care professionals and people with CLBP expressed concern about the validity of automatically-collected data, its security, and the individual’s privacy.

The discussions went well beyond the functionality of the device. The results highlighted the importance of developing an intervention around the mHealth device to help people with CLBP manage their activity and pain, rather than simply relying on the mHealth device. Human support was stated as an essential component of long-term care for people with CLBP. These results confirmed the findings of Svendsen et al [66] that technology alone was not enough to encourage people to engage in self-management. All participants were very clear about the conditions in which the device should be made available, that is, its implementation should be supported by a health care professional, and education should be provided on the importance of physical activity, its role in pain management, and the operation of the device. Education is important to increase the effectiveness of device use because people’s beliefs determine their behavior [67]. These findings go beyond those of the pilot study by Ellingson et al [68], which showed the promising effects of a device-based intervention combined with minimal human support for people with CLBP; because they specified the expected characteristics of human support, in particular, the context in which the device should be offered, how it should be supported by health care professionals, and the frequency at which health care professional support should be provided.

After validating the needs of people with CLBP and health care professionals regarding mHealth devices, we developed design recommendations using the Behavioral Intervention Technology model (Figure 2) [56]. This model helped to structure the needs identified during the 3 phases of the study and to link them to the behavior change strategies that could be integrated within a new intervention encompassing the use of an mHealth device. These design recommendations should now be further developed in the light of behavior change theories and taxonomies currently published in the literature (eg, Michie’s taxonomy) [14,69,70]. Future research should also seek to discuss and refine the prototype development phase with end users. Indeed, after this initial conceptualization phase, this synthesis could be revisited at a later stage to continue the prototype development phase, still using a user-centered approach.

### Limitations

This study was based on the experiences of experts and potential end users. It first identified the needs of 9 experts in phase 1, followed by 26 people with CLBP and health care professionals in phase 2, and then validated these needs with a large number of participants (86 in the first round and 62 in the second round). Despite the sample size, the first limitation concerns the population studied, and its impact on the representativeness and transferability of the results. The participants with CLBP and the health care professionals were all involved in multidisciplinary rehabilitation; therefore, the results may not be generalizable to other settings. This choice was made to have a comparable, homogeneous sample, but the needs and experiences of people with CLBP who do not have access to multidisciplinary rehabilitation (ie, only physiotherapy sessions) may be different.

The Delphi method used in phase 3 may have been subject to some bias. Although the needs for device design were developed during phases 1 and 2, not all the elements that emerged in the first 2 phases were specifically examined during phase 3. The elements selected by the research team for the Delphi questionnaire are those that have been the subject of significant debate among the participants in the previous phases. Thus, in phase 3, particular attention was paid to the variables collected by the device, the content, and the condition of use. This process may have left out elements that would have been also important to present in the Delphi questionnaire. In addition, participants involved in phase 2 were invited to take part in phase 3, which might have introduced bias because of their previous involvement in defining the needs. This involvement may have resulted in an overestimation of the consensus level achieved in the 2 rounds of Delphi questionnaires.

### Conclusions

This study used a UCD approach to explore the needs of people with CLBP and health care professionals regarding the use of a device to support self-managed physical activity. It identified potential users’ expectations regarding the device’s objectives, behavior-change strategies supported by the device, the device’s functionalities, its technical features, and facilitators and barriers that may influence its implementation in a clinical context. The results were used to delineate design recommendations for the development of an mHealth device specifically for people with CLBP and health care professionals. These recommendations will be used in the future to prototype innovative devices that could be offered by health care professionals to people with CLBP in a follow-up rehabilitation context.

## Appendix

Multimedia Appendix 1 Characteristics of people with chronic low back pain and healthcare professionals who participated in each phase of the study.

## Abbreviations

CLBP: chronic low back pain
mHealth: mobile health
UCD: user-centered design
