# Supplementary material for: Exploring the Needs of People With Chronic Low Back Pain and Health Care Professionals for mHealth Devices to Support Self-Managed Physical Activity and Pain: User-Centered Design Approach
Source: JMIR Hum Factors. 2024 Nov 7;11:e59897. doi: 10.2196/59897 (PMC11582481; doi:10.2196/59897)
Supplement: Multimedia Appendix 1 [file humanfactors_v11i1e59897_app1.docx]

## Multimedia Appendix 1

Table 1: Characteristics of participants with CLBP in each phase of the study.

|  | Phase 1  (n = 2)  n (%) | Phase 2  (n = 11)  n (%) | Phase 3 round 1  (n = 45) n (%) | Phase 3 round 2  (n = 33) n (%) |
| --- | --- | --- | --- | --- |
|  |  |  |  |  |
| Gender |  |  |  |  |
| Woman | 2 (100) | 8 (73) | 35 (78) | 27 (82) |
| Man | 0 (0) | 3 (27) | 9 (20) | 5 (15) |
| Other | 0 (0) | 0 (0) | 1 (22) | 1 (3) |
| Age (years) |  |  |  |  |
| 20 – 29 | 0 (0) | 0 (0) | 2 (4) | 2 (6) |
| 30 – 39 | 0 (0) | 1 (9) | 6 (13) | 5 (15) |
| 40 – 49 | 0 (0) | 3 (27) | 10 (22) | 6 (18) |
| 50 – 59 | 2 (100) | 5 (46) | 19 (42) | 12 (36) |
| ≥ 60 | 0 (0) | 2 (18) | 8 (18) | 8 (24) |
| Location |  |  |  |  |
| Switzerland | 1 (50) | 4 (36) | 39 (87) | 27 (82) |
| France | 1 (50) | 7 (64) | 6 (13) | 6 (18) |

Table 2: Characteristics of healthcare professionals who participated in each phase of the study.

|  | Phase 1  (n = 3)  n (%) | Phase 2  (n = 15)  n (%) | Phase 3 round 1  (n = 41)  n (%) | Phase 3 round 2  (n = 28)  n (%) |
| --- | --- | --- | --- | --- |
|  |  |  |  |  |
| Gender |  |  |  |  |
| Woman | 1 (33) | 8 (53) | 11 (27) | 9 (32) |
| Man | 2 (67) | 7 (47) | 30 (73) | 19 (68) |
| Other | 0 (0) | 0 (0) | 0 (0) | 0 (0) |
| Age (years) |  |  |  |  |
| 20 – 29 | 0 (0) | 1 (7) | 6 (15) | 1 (4) |
| 30 – 39 | 1 (33) | 5 (33) | 14 (34) | 9 (32) |
| 40 – 49 | 0 (0) | 6 (40) | 7 (17) | 6 (21) |
| 50 – 59 | 2 (67) | 3 (20) | 11 (27) | 10 (36) |
| ≥ 60 | 0 (0) | 0 (0) | 3 (7) | 2 (7) |
| Location |  |  |  |  |
| Switzerland | 2 (67) | 7 (4) | 14 (34) | 13 (46) |
| France | 1 (33) | 8 (53) | 13 (32) | 15 (54) |
| Profession |  |  |  |  |
| Occupational therapist | 1 (33) | 4 (27) | 7 (17) | 4 (14) |
| Physiotherapist | 0 (0) | 5 (33) | 17 (42) | 11 (39) |
| Physical activity educator | 0 (0) | 2 (13) | 3 (7) | 2 (7) |
| Medical doctor | 2 (67) | 3 (20) | 13 (32) | 11 (39) |
| Psychologist | 0 (0) | 1 (7) | 0 (0) | 0 (0) |
| Osteopath | 0 (0) | 0 (0) | 1 (2) | 0 (0) |
| CLBP experience (years) |  |  |  |  |
| < 5 | 0 (0) | 6 (40) | 9 (22) | 5 (18) |
| 5 – 10 | 0 (0) | 5 (33) | 7 (17) | 6 (21) |
| 11 – 20 | 1 (33) | 1 (7) | 17 (42) | 11 (39) |
| 21 – 30 | 2 (67) | 3 (20) | 6 (15) | 5 (18) |
| > 31 | 0 (0) | 0 (0) | 2 (5) | 1 (4) |
